# Borderline Personality Disorder in Adolescence as a Generalization of Disorganized Attachment

**DOI:** 10.3389/fpsyg.2018.01962

**Published:** 2018-10-16

**Authors:** Raphaële Miljkovitch, Anne-Sophie Deborde, Annie Bernier, Maurice Corcos, Mario Speranza, Alexandra Pham-Scottez

**Affiliations:** ^1^Laboratoire Paragraphe EA 349, Paris 8 University, Saint-Denis, France; ^2^Department of Psychology, University of Montreal, Montreal, QC, Canada; ^3^Adolescent Psychiatry Department, Institut Mutualiste Montsouris, Inserm U 669, Faculty of Medecine, University René Descartes-Paris V, Paris, France; ^4^Inserm U 669, Faculty of Medecine, University René Descartes-Paris V, Paris, France; ^5^Child Psychiatry Department, Versailles Hospital, Versailles, France; ^6^Unité de Recherche EA4047, Recherches Cliniques et en Santé Publique sur les Handicaps Psychique, Cognitif et Moteur (HANDIReSP), Université de Versailles Saint-Quentin-en-Yvelines, Versailles, France; ^7^Inserm U 669, CMME, Sainte-Anne Hospital, Paris, France

**Keywords:** borderline personality disorder, adolescence, representations, internal working models, attachment, disorganized attachment, generalization

## Abstract

Several researchers point to disorganized attachment as a core feature of borderline personality disorder (BPD). However, recent studies suggest that specific internal working models (IWMs) of each parent combine to account for child outcomes and that a secure relationship with one parent can protect against the deleterious effects of an insecure relationship with the other parent. It was thus hypothesized that adolescents with BPD are more likely to be disorganized with both their parents, whereas non-clinical controls are more secure with at least one of their caregivers. Thirty-six adolescents with BPD and 30 control participants (aged 13–19) were included. Psychiatrist diagnosis was verified with the Structured Interview for DSM-IV Personality Disorders (SIDP-IV) and comorbidity was assessed using the Kiddie-SADS. Reported trauma was assessed with the Childhood Trauma Questionnaire (CTQ). Attachment IWMs of each parent were assessed with the Attachment Multiple Model Interview (AMMI), which enables separate coding for each attachment figure and in which disorganization is conceptualized as conflicting attachment strategies within a specific relationship. Results of a logistic regression analysis suggested that beyond insecure attachment, being disorganized not just with one but with both parents is particularly characteristic of adolescents with BPD. Conversely, belonging to the non-clinical group was predicted by higher security scores with the father and lower deactivation with the mother. Although higher levels of childhood abuse or neglect were reported by adolescents with BPD, the retained attachment dimensions predicted group membership over and above reported trauma. These findings have important implications for clinical intervention and highlight the protective role fathers may have.

## Introduction

Borderline personality disorder (BPD) is one of the few personality disorders considered to be of special interest in adolescence (Tyrer, 2014). BPD is usually first diagnosed in adolescence (Chanen and Kaess, 2012) but symptoms may wane in early adulthood if youths receive psychological assistance (Schuppert et al., 2009; Chanen and McCutcheon, 2014). Therefore, early identification of factors involved in the disorder is important to prevent symptoms from setting in durably (Chanen and McCutcheon, 2014; Sharp and Fonagy, 2015).

Borderline symptomatology fluctuates as a function of the vicissitudes of relationships (American Psychiatric and Association, 2013). Adolescence is particularly critical in this respect because learning to regulate affect “in and through” social interactions becomes a central task during this developmental stage (Allen and Manning, 2007). At the same time, the struggle for autonomy is thought to reactivate unresolved attachment-related issues (Allen and Miga, 2010). Neurobiological developmental changes may further challenge psychologically or genetically vulnerable teenagers (Cicchetti and Rogosch, 2002). According to Sharp and Fonagy (2008), insecure attachment is a major predisposing factor for BPD as it interacts with the normative developmental changes and challenges of adolescence. According to Liotti (2014), individuals with disorganized attachment would be at particular risk for the disorder. In the present study, we examine whether the onset of BPD is more likely when adolescents are highly disorganized not just with one but with both parents, and whether security provided by at least one parent may on the contrary be associated with a reduced risk of having the disorder.

### Attachment in BPD

Numerous parallels can be drawn between attachment disorganization and BPD. First, borderline features echo those of disorganized attachment (Holmes, 2004; see also Gunderson, 2007): (1) disorganization is defined as conflicting attachment strategies (e.g., approach and avoidance) toward a caregiver, who represents both a secure base and a source of threat (Main and Solomon, 1986). Likewise, one of the main criteria for BPD is instability in interpersonal relationships, with dramatic shifts from idealization to devaluation of others (American Psychiatric and Association, 2013); (2) like BPD, disorganization is thought to involve a fragmented or unstable sense of self (Liotti, 2004; Beebe et al., 2010); (3) disorganized attachment is associated with physiological disturbances in the regulation of stress (Bernard and Dozier, 2010). Emotional dysregulation is also a central characteristic of BPD (Putnam and Silk, 2005); (4) associations between disorganized attachment and features of BPD such as impulsive (Jacobsen et al., 1997) or dissociative symptoms (Ogawa et al., 1997) have been reported (see Lyons-Ruth, 2008 for a review); (5) disorganized infants’ experience of “fright without solution” (Main, 1995) is associated with feelings of insecurity and caregiver inaccessibility (Lyons-Ruth et al., 1999; see also George and Solomon, 2008 for a review). Likewise, borderline patients experience intense fear of loss and abandonment.

In addition to common characteristics, it has been proposed that attachment disorganization could be a risk factor in the development of the disorder. Main and Hesse (1990) proposed that in disorganized dyads, rapid shifts in the caregiver’s behavior lead young children to develop multiple (contradictory) models of self and other (see also Beebe et al., 2010). These contradictory models are believed to explain why disorganized infants exhibit conflicting behavioral strategies. According to Liotti (2014), such unintegrated models render children vulnerable to dissociation in the face of further traumatic experiences and lead to dysregulated affects and impulses. Hence, he posited that disorganized attachment is at the core of borderline instability (see also Bo et al., 2015).

In line with this view, Lyons-Ruth et al. (2005) found that borderline features at age 19 were predicted by disrupted maternal affective communication in infancy, which itself is associated with disorganized attachment (Madigan et al., 2006). Disorganized/controlling behavior at age 8 was also associated with borderline features in late adolescence (Lyons-Ruth et al., 2013). However, only one participant from this longitudinal sample actually met criteria for diagnosis. Similarly, Carlson et al. (2009) found disorganized attachment (with the mother) at 12 and 18 months to be associated with borderline symptoms at age 28. Although participants were from a high-risk sample, the symptom counts provided do not enable determination of the actual number of diagnosed borderline adults in the sample.

Concurrent data on adult clinical samples also support the hypothesis of disorganized attachment as being associated with BPD. Patrick et al. (2008) developed an alternative system for coding the Adult Attachment Interview (AAI), designed to assess contradictory representations of caregiver and self (hostile/helpless state of mind). The hostile/helpless state of mind is akin to the notion of contradictory segregated models proposed to be central to disorganized attachment (Main and Hesse, 1990). On a sample of 12 borderline women, Lyons-Ruth et al. (2007) found that all participants displayed hostile/helpless states of mind. However, comorbidity, which is very common in BPD (Ha et al., 2014), was screened out. In addition to the small sample size, this screening of participants limits generalization of the results. Nevertheless, this study suggests that multiple (contradictory) models of attachment in adulthood may be a feature of BPD.

Several cross-sectional adult studies using the AAI (George et al., 1985) show that patients with BPD more often display unresolved or fearfully preoccupied states of mind (Barone et al., 2011; Buchheim and George, 2011; see Bakermans-Kranenburg and van IJzendoorn, 2009 for a meta-analysis; see also Rosenstein and Horowitz, 1996 and Kobak et al., 2009 for similar findings with adolescents). The unresolved category is understood as revealing disorganized attachment because it suggests a collapse of strategy. This is inferred from lapses in reasoning or discourse when recounting past experiences of trauma or loss. The preoccupied category, however, does not correspond to disorganization. In the “fearfully preoccupied” subcategory, which is frequently observed among borderline patients, interviewees report fearful experiences, which are presently preoccupying or even “unpredictably controlling mental processes” (Main et al., 2002).

Thus, what is common to both the unresolved and the fearfully preoccupied categories is reported experience of traumatic events. A great deal of research has shown strong associations between BPD and trauma or abuse (e.g., Battle et al., 2004). It is therefore possible that the unresolved and fearfully preoccupied states of mind observed in patients with BPD are confounded with the experience of trauma. However, in the above studies using the AAI, the effect of trauma *per se* was not controlled for. Therefore, it cannot be concluded that unresolved or fearfully preoccupied states of mind, rather than actual trauma, are associated with the emergence of BPD.

### Combined Effects of IWMs of Each Parent

Unlike disorganized attachment whose features appear specifically in relation to the caregiver, borderline symptomatology is pervasive and occurs in a variety of contexts. In this respect, BPD could perhaps reflect a generalization of the attachment pattern developed within the relationship with a specific caregiver. Abundant research suggests that internal working models (IWMs) of relationships with parents constitute a major lens through which other interactions are perceived (e.g., Dykas et al., 2012). Additional research also suggests that specific IWMs of each parent combine to account for child outcomes. For instance, two studies found that children who had secure attachments to both their parents showed superior social skills, followed by those who had only one secure attachment, and finally by those with insecure attachments to both their parents (Suess et al., 1992; Verschueren and Marcoen, 1999). More recently, Boldt et al. (2014) observed that among children who were insecure with their mother, variations in security with the father had significant implications for adaptation (see also Kochanska and Kim, 2013).

Closer to our purpose, in the Zanarini et al. (1997) sample of borderline patients, 92% reported bi-parental neglect and emotional denial (see also Zanarini et al., 2000). According to Fonagy et al. (2003), even a single experience of a secure/understanding relationship may be sufficient for the development of reflective processes, which help integrate contradictory IWMs resulting from trauma, and hence foster better adaptation. Presumably then, consideration of relationships with both parents can be expected to provide more insight into overall patterns of relatedness in adolescents than a focus on the mother only. Although research has documented attachment states of mind associated with BPD (Bakermans-Kranenburg and van IJzendoorn, 2009; Barone et al., 2011; Buchheim and George, 2011), knowledge is limited when it comes to understanding how specific attachment models of each parent combine to explain the disorder. Because attachment in adolescence and adulthood has been conceptualized in terms of general states of mind in developmental research, relationship-specific models associated with BPD have not been examined. But recent research suggests that the development of an overall state of mind with respect to attachment does not preclude the continuing existence of relationship-specific representations (Miljkovitch et al., 2015).

The Attachment Multiple Model Interview (AMMI; Miljkovitch, 2009; Miljkovitch et al., 2015) was designed to provide separate assessments of IWMs for each attachment relationship. In this respect, it differs from certain conceptualizations of adolescent or adult attachment, namely the AAI, which assesses overall states of mind with no distinction between mother and father. Although the AAI includes specific “experience” scales for each parent, classifications are assigned on the basis of state of mind scales which refer to attachment in general (Main et al., 2002). Hence, the AAI “Cannot Classify” category is assigned when interviewees display mixed states of mind. Because the coexistence of different states of mind is assumed to reflect contradictory models, the CC category is considered to reflect disorganization (with a more global breakdown than in the unresolved category). Note however that this classification is also assigned when “the speaker presents different states of mind in describing different people” (p. 190). Disorganization according to the AMMI is also assumed to capture contradictory models, but in line with Main and Solomon’s (1986) original definition of disorganization, it considers the coexistence of conflicting attachment strategies *within* a relationship, and does so not only at the level of behavior, but at the level of representation as well. Because factors other than abuse or loss can be involved in disorganized attachment (Lyons-Ruth et al., 1999; Grienenberger et al., 2005; Hesse and Main, 2006; Madigan et al., 2006; Miljkovitch et al., 2013), disorganization according to the AMMI does not require that interviewees report such experiences (as opposed to the AAI “unresolved” classification). This has the advantage of disentangling disorganized functioning *per se* from its potential causes.

Assuming that the pervasive pattern of instability in interpersonal relationships among adolescents with BPD reflects a generalized inability to represent relationships according to a consistent, stable model, more disorganized attachment models of relationships with both primary caregivers can be expected. We thus hypothesized that BPD among adolescents is associated with more disorganized attachment models of both primary caregivers. Given the protective function of secure attachment, we expected a non-clinical control group to have higher scores of security with at least one parent. These results are expected to be found even after reported trauma has been controlled for.

## Materials and Methods

### Participants and Procedure

One group of borderline patients and one group of non-clinical adolescents were recruited. The samples were drawn from a European research project (European Research Network on BPD, EURNET BPD) investigating correlates of BPD in adolescence (13–19 years). The research network involved five academic psychiatry departments specialized in adolescents and young adults in France, Belgium, and Switzerland. This study was carried out in accordance with the recommendations of the French National Committee for Personal Freedoms with written informed consent from all participants. All participants, and at least one of their parents for those under 18 years of age, gave written informed consent in accordance with the Declaration of Helsinki. The protocol was approved by the French National Committee for Personal Freedoms.

Borderline participants were recruited in these psychiatry departments (inpatient and outpatient hospital units). Patients aged 13–19 were considered for inclusion if they presented BPD according to their psychiatrist. Patients with psychotic disorders were excluded from the study for feasibility reasons. BPD and other diagnoses were assessed with semi-structured interviews (see Section “Measures” below). The diagnostic interviews were conducted by a team of five clinical psychologists and psychiatrists experienced in the assessment of DSM-IV Axis I and II disorders in adolescents. After this screening procedure, the patients whose diagnosis of BPD was confirmed were interviewed in their respective psychiatry departments. Because the number of male volunteers was too small and because borderline symptomatology varies according to gender (Johnson et al., 2003), the decision to include only female participants was made at an early stage of the study. The final sample included 36 outpatients.

Control group participants were recruited using advertisement for the study in schools and universities knowing that participants from the clinical group were mostly from upper middle class backgrounds (see Section “Descriptive Statistics” below) and were still in school. The procedure with the non-clinical sample was identical to that with the borderline sample. Control participants were screened in order to make sure they did not have BPD [according to the Structured Interview for DSM-IV Personality Disorders (SIDP-IV)] or current or lifetime mental disorders (according to the Kiddie-SADS). For better contrast, adolescents who had consulted a psychiatrist or psychologist were also excluded from the study control group. Thirty control participants from similar socioeconomic backgrounds were thus included.

### Measures

#### Borderline Personality Disorder

The SIDP-IV (Pfohl et al., 1997) was used to confirm BPD diagnosis among patients and screen for personality disorders among all participants. Borderline severity for each of the nine criteria was coded as absent (0), subthreshold (1), present (2), or prominent (3). Borderline severity scores thus varied from 0 to 27. The clinical threshold is 10 (with scores of at least 2 on at least 5 of the nine scales). The SIDP-IV has shown good psychometric properties on adolescent and young adult samples (Chabrol et al., 2002). The inter-rater reliability for SIDP-IV was calculated from independent ratings of ten videotaped interviews. The Kappa coefficient for agreement on the presence or absence of BPD was very high (0.84) and the values for the presence/absence of the other personality disorders ranged from 0.54 to 1.

#### Psychiatric Diagnoses

The Kiddie-SADS (Kaufman et al., 1996; see Kaufman et al., 1997 for data on psychometric properties) was used to verify the absence of psychiatric disorders among control participants and to assess psychiatric comorbidity among patients. Diagnoses were established according to DSM-IV criteria. To obtain high levels of reliability, the research team participated in several training sessions, including the commented scoring of videotaped interviews and a training session conducted by the developers of the Kiddie-SADS (Boris Birmaher and Mary Kay Gill). Final diagnoses were established by the best estimate method on the basis of the interviews and any additional relevant data from the clinical record according to the LEAD standard (Pilkonis et al., 1991).

#### Socio-Demographic Information

A brief *ad hoc* self-report questionnaire was administered in order to ensure that control participants had never consulted for a psychiatric disorder, and to obtain socio-demographic data (i.e., parental employment status and education). Parental SES was determined according to each parent’s employment status. A dimensional index was developed as follows: (1) low-grade salary workers, (2) intermediate professions, and (3) white collar workers. Parental education was coded according to three levels of education: (1) technical training, (2) secondary education, and (3) higher education. Mean scores of mother and father SES as well as education were used.

#### Childhood Trauma

To control the possible confounding effect of reported experiences of abuse, the Childhood Trauma Questionnaire (CTQ, Bernstein and Fink, 1998) was administered. It is a 28-item self-report inventory that provides brief, reliable, and valid screening for histories of abuse and neglect. It inquires about five types of maltreatment: emotional, physical, and sexual abuse, and emotional and physical neglect. Because of the small number of participants, we tried to limit the number of predictors so as to diminish the probability of Type-II error. Therefore, we did not use the CTQ subscales separately, and opted for the global score (i.e., the sum of scores for each type of maltreatment).

#### Attachment Models

The AMMI (Miljkovitch et al., 2015) is a semi-structured interview in which participants are asked to describe their emotional and behavioral reactions with each attachment figure in a variety of attachment-related situations (separation, illness, stress, danger, conflict, etc.). It was designed to assess specific models of attachment in adolescence or adulthood, that is, how a specific relationship has been internalized (as more or less secure) as well as the attachment strategies resulting from this internalized representation (orienting attention and behavior toward or away from attachment). Deactivation in the AMMI refers to a turning away from attachment, whether mentally and/or behaviorally. Hyperactivation refers to a focus on the attachment figure and more or less explicit attempts to elicit his/her attention.

In accordance with recent work suggesting that the latent structure of individual differences in IWMs is consistent with a continuous rather than a clear-cut taxonomic model (Fraley and Roisman, 2014), the AMMI provides continuous scores on each attachment dimension (security, deactivation, hyperactivation, and disorganization). Security, deactivation, and hyperactivation were coded on continuous 9-point scales (from 0 to 8). Following Main and Solomon’s (1986) original definition of disorganization as conflicting attachment strategies, disorganization is assigned when participants exhibit both deactivation and hyperactivation within the same relationship (see Miljkovitch, 2009; see also Beeney et al., 2016 for a similar rationale). The more these opposite strategies coexist, the higher the disorganization score (see Miljkovitch et al., 2015); scores vary from 0 to 16.

The validity of the AMMI has been established with longitudinal data gathered from age 4 to 23, showing that AMMI scores at age 23 reflect corresponding cumulated lifetime scores for security, deactivation, and hyperactivation. A significant link between AMMI disorganization with mother and AAI unresolved trauma has also been observed (Miljkovitch et al., 2015).

Participants were asked to describe their relationship with each parent. Because many of the participants did not have romantic partners, they were not interviewed on this type of relationship. All AMMI verbatim transcripts were coded by two blind coders trained by the developer of the instrument. When all the dimensions were considered, differences of less than 1 point were found for 86% of the codings. Disagreements were discussed and reconciled. Scores assigned after consensus were then compared with the coding of a third blinded coder. Differences of 1 point or more were then found for 7% of the codings, and were discussed in order to determine final scores.

### Statistical Analyses

To determine which variables were likely to predict BPD, *t*-tests comparing means of attachment and trauma for the BPD and the control group were run. Those variables on which group differences were found were included in a logistic regression analysis predicting BPD. Because we hypothesized that disorganization toward both parents is associated with BPD, the interaction term between these two variables was also entered as a predictor. Beforehand, preliminary analyses were run to determine whether any sociodemographic variables needed to be controlled for. Student *t*-tests were thus performed to identify differences in terms of SES, parental education, and age between the two groups. Correlations between these sociodemographic variables and each attachment scale were also conducted. Because most patients with BPD also had at least one additional Axis I or Axis II disorder, comparisons between those who were diagnosed with a specific comorbid disorder and those who were not were conducted to examine the possible effects of these other disorders on the AMMI dimensions.

## Results

### Descriptive Statistics

Many borderline patients exhibited additional personality disorders: non-exclusively, 43% obsessive-compulsive, 27% avoidant, 13% paranoid, 10% antisocial, 7% histrionic, 7% dependent, 3% narcissistic, and 3% schizotypal personality disorders. Co-occurring Axis 1 disorders were also frequently diagnosed, the two most common being eating (58%) and mood disorders (42%), followed by anxiety (14%) and substance use disorders (8%) (see Ha et al., 2014 for similar findings). The mean score on the SIDP-IV was 16.70 (*SD* = 1.20) in the BPD group, compared with 2.07 (*SD* = 0.42) in the control group.

### Preliminary Analyses

Student *t*-tests revealed no differences between the two groups for SES (*M_controls_* = 2.48, *SD* = 0.51; *M_patients_* = 2.30, *SD* = 0.71; *t =* 1.06; *p* = 0.29), parental education (*M_controls_* = 2.45, *SD* = 0.56; *M_patients_* = 2.31, *SD* = 078; *t =* 0.85; *p* = 0.40), or age (*M_controls_* = 16.28, *SD* = 1.03; *M_patients_* = 16.80, *SD* = 1.06; *t =* 1.92; *p* = 0.06). Age was the only potential covariate that was significantly correlated with one of the attachment variables (i.e., hyperactivation toward the mother: *r* = 0.31, *p* = 0.02).

When comparing patients with non-clinical participants, expected differences were found on all the AMMI dimensions, whether in relation to the mother or the father (i.e., borderline patients were given lower security scores and higher deactivation, hyperactivation, and disorganization scores). CTQ scores were also significantly higher in the borderline group (see **Table 1**).

**Table 1:** Mean scores, standard deviations, and ranges for the AMMI and CTQ dimensions according to group membership.

	Controls			Patients			*t*
	*M*	*SD*	Range	*M*	*SD*	Range	
Security mother	6.80	1.83	[0-8]	3.54	1.88	[0-7]	7.15^∗∗∗^
Deactivation mother	2.20	2.25	[0-8]	6.24	1.40	[2-8]	-8.99^∗∗∗^
Hyperact. mother	2.20	1.83	[0-5]	4.73	2.29	[0-8]	-4.91^∗∗∗^
Disorg. mother	2.73	2.75	[0-8]	8.91	4.31	[0-14]	-7.12^∗∗∗^
Security father	6.30	1.93	[1-8]	2.39	1.81	[0-6]	8.48^∗∗∗^
Deactivation father	2.60	2.30	[0-8]	6.53	1.58	[2-8]	-8.20^∗∗∗^
Hyperact. father	1.53	1.78	[0-6]	3.42	2.49	[0-8]	-3.47^∗∗^
Disorg. father	1.87	2.29	[0-6]	6.72	4.95	[0-16]	-5.25^∗∗∗^
CTQ total score	6.43	2.29	[5-17]	9.51	2.49	[6-17]	-4.91^∗∗∗^

*^∗∗^p < 0.01, ^∗∗∗^p < 0.001.*

No effect of comorbidity (i.e., eating, mood, and anxiety disorders, obsessive-compulsive and avoidant personality disorders) was found on any of the AMMI dimensions with either parent (all *t*s < 1.67, *ns*). However, group comparisons could not be performed for disorders for which sample sizes were too small (*Ns <* 5).

### Main Analyses

The CTQ, the AMMI dimensions, and the interaction term between disorganization with the mother and disorganization with the father were entered as predictors in a logistic regression analysis with BPD as the dependent variable. The backward method was chosen so that the respective contribution of each variable would be considered during the selection process. The analysis (see **Table 2**) retained the CTQ score (OR = 1.55; Wald test = 4.90), deactivation with the mother (OR = 1.75; Wald test = 4.34), security with the father (OR = 0.54; Wald test = 6.55), and the interaction term between disorganization with the mother and with the father (OR = 1.06; Wald test = 2.36) as predictors of BPD. The total model explained 86.9% of the variance and led to a correct classification of 93.9% of the participants (28/30 in the control group and 34/36 in the BPD group).

**Table 2:** Logistic regression predicting borderline personality disorder (backward method).

Variable	Model log likelihood	Change in -2 log likelihood	*df*	Sig. of the change
CTQ	-13,872	6,118	1	, 013
Deactivation mother	-14,313	6,999	1	, 008
Security father	-15,693	9,759	1	, 002
Disorganization mother	-15,740	9,855	1	, 002
^∗^Disorganization father				

To describe the moderating effect of disorganization toward the father more precisere precisely, we divided the whole sample into three groups of equivalent sizes according to scores of disorganization with the mother (<4, between 4 and 9, and >9). We then calculated for each group partial point-biserial correlations between disorganization with the father and BPD, controlling for the CTQ. For the group with low scores of disorganization toward the mother, the correlation was close to zero: *_part._r* = 0.02, p = 0.93. For the intermediate group, the correlation was higher and significant: *_part._r* = 0.57, p = 0.01. For the group of participants whose scores were above 9, the correlation was equal to 1 (i.e., all had BPD). In other words, disorganization with the father progressively increased the chances of having BPD as disorganization with the mother increased also. The same procedure was followed by making groups according to disorganization with the father (=0, from 1 to 5, and >5). Results were similar but the differences between the correlations were not as big with *_part._r* = 0.14, p = 0.54 for the group with low scores, *_part._r* = 0.36, p = 0.19 for the intermediate group, and *_part._r* = 0.54, p = 0.005 for the group with the highest scores.

## Discussion

The aim of the present study was to examine disorganized attachment as a factor involved in BPD in adolescence. Assuming that when disorganized attachment models accumulate, they are more likely to be pervasive and to be associated with a generalized pattern of instability, we expected adolescents with BPD to exhibit higher disorganization with both parents than control adolescents. We also expected the controls to be more secure with at least one parent. Comparisons between the two groups confirmed these hypotheses. In fact, the statistical analysis revealed that over and above what was accounted for by reported trauma, what best distinguished borderline from control adolescents was a combination of disorganization toward each parent, insecurity toward the father, and deactivation of the attachment system in the relationship with the mother.

The fact that borderline patients were more likely to have disorganized models of their parents echoes the results of Lyons-Ruth et al. (2007), based on the hostile/helpless classification, and points to contradictory attachment models as a core feature of BPD. Although AMMI disorganization is inferred on the basis of high levels of deactivation and hyperactivation within the same relationship, it is interesting to note that findings with this interview are similar to those found with an instrument (the HH system) explicitly designed to assess unintegrated attachment models. This observation is consistent with Main and Hesse’s (1990) assumption that disorganization reflects contradictory representations of the attachment figure. Recently, Beeney et al. (2016) also found borderline severity to be associated with “disorganized-oscillating” attachment among psychiatric outpatients.

In addition to showing that contradictory models of caregivers are more common among patients with BPD than healthy controls, our findings suggest that being disorganized with not just one, but with both parents, is particularly characteristic of borderline adolescents. This is coherent with the report by Zanarini et al. (2000) of particularly high rates of bi-parental abuse or neglect among borderline patients. Although disorganization toward the mother may constitute a risk factor for the development of borderline symptoms (Carlson et al., 2009), one way of interpreting the present findings is to consider that the putative deleterious effects of disorganization with the mother may be increased when the relationship with the father also leads to a disorganized model. It seems reasonable to think that a “corroboration” in the relationship with the father of the model resulting from interactions with the mother (or *vice versa*) may place the adolescent at greater risk of generalizing his/her IWMs to other relationships, and thus of developing a rigid and pervasive pattern of relatedness, as is the case in BPD.

Conversely, when disorganization toward one parent is counterbalanced by organized and especially secure attachment toward the other parent, the generalization of instability is less likely. Bowlby (1980) contended that divergent interpersonal input could render IWMs more flexible and open to change. In such cases, the organized relationship may provide an alternative to the disorganized model, offering the opportunity for more stable functioning. Fonagy et al. (2003) also proposed that by fostering the capacity for mentalization, a secure relationship offers the opportunity to integrate incoherent models of experience and thereby reduces their negative impact on psychological adjustment. Our findings are also consistent with previous studies showing that a secondary secure strategy may dampen the deleterious effects of disorganized attachment (e.g., Luijk et al., 2010).

In addition to the findings on disorganized models, deactivation toward the mother also significantly differentiated the two groups. According to Linehan (1993), BPD develops as a result of emotional dysregulation, which occurs when emotional vulnerabilities are responded to in “invalidating” ways. It can be assumed that deactivation of the attachment system reflects an internalization of this inability to share true emotional experience with parents (and eventually with the self). Deactivation in the relationship with the mother was found to be more predictive of BPD than deactivation toward the father. Many studies on the mediating role of maternal conversational style (e.g., Peterson and Slaughter, 2003; see also Bretherton, 1993), mind-mindedness (Meins et al., 2002), and reflective function (Fonagy and Target, 1997) point to the mother’s major contribution to her child’s developing socio-cognitive understanding. Thus, it is not surprising that restricted communication in the relationship with the mother (as suggested by deactivation of the attachment system) may act as a serious obstacle for healthy personality growth. Nevertheless, more research is needed to understand the specific role of fathers in healthy or abnormal socio-cognitive development.

In the present study, although higher levels of childhood abuse or neglect were reported by borderline adolescents, the selected attachment representations predicted group membership over and above what these reported events accounted for. This constitutes an extension of what Zanarini et al. (2000) called “bi-parental failure in the childhood experiences of borderline patients,” in that beyond actual life events, what seems most deleterious is an internalization of the impossibility of finding support from caregivers (insecurity and deactivation) and of obtaining help in “metabolizing” traumatic experience.

The logistic regression also revealed that in combination with disorganized models and deactivation of the attachment system in relation to the mother, insecurity toward the father was a significant predictor of BPD. This finding is in line with previous work (Bakermans-Kranenburg and van IJzendoorn, 2009; Barone et al., 2011) suggesting that insecure attachment is the rule, rather than the exception, among borderline patients. Interestingly however, it was security toward the father that best differentiated borderline from control adolescents. Borderline adolescents were also less secure than controls toward their mothers, but when considered in combination with the other predictive variables, this finding – unlike security toward the father – was no longer significant. During the AMMI, many borderline patients reported abusive and out-of-control paternal behavior, which was less often the case in the relationship with the mother (see Zanarini et al., 2000 for similar findings). Therefore, more intense experiences of insecurity with the father might account for these findings. Security scores with the father were indeed lower than those with the mother (see **Table 1**). However, because this interpretation partly relies on anecdotal observations from our interviewers, systematic research is needed to confirm this tentative explanation. Also, the fact that non-clinical adolescents displayed more secure models of their relationship with their fathers highlights the protective function this parent may have. Overall, the present study points to the need for a more systemic approach: examining attachment beyond the mother–child dyad brings a fuller understanding of the dynamics at play in the construction of IWMs, and of what determines their scope of influence.

An alternative explanation could also account for the findings, as the direction of the effect may be the reverse of that considered above. Rather than showing that the generalized pattern of instability among borderline adolescents results from the accumulation of conflicting models, it is possible that the disorder distorts their attachment representations, leading them to describe (and perhaps experience) all relationships in accordance with their present functioning. Longitudinal research is still needed to test the hypothesis of BPD as an “outgrowth” of disorganized attachment. In any case, the present study corroborates the assumption that attachment disorganization is a core feature of BPD, extends this observation to a clinical sample with confirmed diagnosis, and calls attention to the need to consider relationships with both parents.

The study has other limitations that must be considered also. First, although it represents a step in the direction of a more systemic approach, relationships likely to impact the construction of IWMs and personality development extend well beyond relationships with parents. Hence, future studies examining relationships with siblings as well as contextual influences outside the family could usefully complement the present data. Likewise, genetic, epigenetic, or constitutional factors involved in the onset of BPD (Gunderson and Lyons-Ruth, 2008; Trerotola et al., 2015; Trull, 2015; Jones-Mason et al., 2016) have not been considered here, and their role should not be minimized.

Second, because control participants were selected as having no psychiatric disorder, the representativeness of this sample is questionable. This procedure was nevertheless preferred so as to obtain greater contrast between the clinical and non-clinical groups and identify borderline specificity more clearly. In future studies, comparisons with other disorders would enable an understanding of the psychopathological pathways specific to BPD.

Although the present clinical sample is quite large compared to those of most studies on borderline adolescents, only female participants were included, thus limiting the generalizability of the results. Future research examining the links between attachment and BPD is still needed to understand the development of BPD among male adolescents. This seems particularly important given that borderline symptomatology varies significantly by gender (Johnson et al., 2003). It is also possible that the impact of attachment to a specific parent is not the same for girls and boys. The sample nevertheless seems representative of female adolescents with BPD in terms of psychiatric co-morbidity (see Kaess et al., 2012).

Axis I and Axis II disorders could have blurred the links between BPD and the attachment measures, but group comparisons between borderline patients with and without the major comorbid disorders revealed no effect of these other diagnoses, and thus provide greater confidence in the specificity of the results regarding attachment among adolescents with BPD. Still, the effects of disorders for which we had very few cases could not be controlled for. Although it seems unlikely that such small subsamples significantly impacted overall tendencies, a possible effect of these other diagnoses cannot be ruled out.

Despite these limitations, the present study has important implications regarding clinical intervention with borderline patients. One major finding is that the selected representational features of attachment account for the disorder over and above reported experiences of abuse or neglect (see also Finger et al., 2015). More specifically, borderline adolescents are more likely to feel insecure, to cumulate contradictory models, and to turn away from attachment. Therefore, psychotherapy aimed at integrating multiple models of specific relationships via the establishment of a secure relationship that fosters the safe exploration of internal states seems particularly recommended (see Holmes, 2001). These findings in an adolescent sample are all the more important because treatment of BPD in that period of life can prevent the disorder from consolidating (Chanen and McCutcheon, 2014). They are also encouraging in view of possible therapeutic intervention: increasing feelings of security and reducing deactivation is in line with the objectives of existing therapies aiming to enhance mindful awareness in the context of a successful therapeutic alliance (e.g., Dialectical Behavior Therapy, Linehan, 1987; Mentalization-Based Treatment, Bateman and Fonagy, 2006; Transference-Focused Psychotherapy, Kernberg et al., 2008). Hence these therapies, originally developed for borderline adults, could presumably be advised for borderline adolescents (see Normandin et al., 2015).

## Author Contributions

RM wrote the manuscript and ran the analyses. A-SD organized collection and coding of the data and helped for manuscript preparation. AB gave expert comments on the manuscript. MC, MS, and AP-S elaborated and coordinated the larger study.

## Conflict of Interest Statement

The authors declare that the research was conducted in the absence of any commercial or financial relationships that could be construed as a potential conflict of interest.

## References

[B1] Allen J. P. Manning N. (2007). From safety to affect regulation: attachment from the vantage point of adolescence. *New Dir. Child Adolesc. Dev.* 117 23–39. 10.1002/cd.192 17876787 PMC3385858

[B2] Allen J. P. Miga E. M. (2010). Attachment in adolescence: a move to the level of emotion regulation. *J. Soc. Pers. Relationsh.* 27 181–190. 10.1177/0265407509360898 20431705 PMC2860752

[B3] American Psychiatric and Association (2013). *DSM-IV-TR: Diagnostic and Statistical Manual of Mental Disorders* 4th Edn. Washington, DC: American Psychiatric Press Inc.

[B4] Bakermans-Kranenburg M. J. van IJzendoorn M. H. (2009). The first 10,000 adult attachment interviews: distributions of adult attachment representations in clinical and non-clinical groups. *Attach. Hum. Dev.* 11 223–263. 10.1080/14616730902814762 19455453

[B5] Barone L. Fossati A. Guiducci V. (2011). Attachment mental states and inferred pathways of development in borderline personality disorder: a study using the adult attachment interview. *Attach. Hum. Dev.* 13 451–469. 10.1080/14616734.2011.602245 21838646

[B6] Bateman A. Fonagy P. (2006). “Mentalizing and borderline personality disorder,” in *The handbook of mentalization-based treatment*, eds Allen J. G. Fonagy P. (Hoboken, NJ: John Wiley & Sons Inc.), 185–200. 10.1093/med/9780198570905.001.0001

[B7] Battle C. L. Shea M. T. Johnson D. M. Yen S. Zlotnick C. Zanarini M. C. (2004). Childhood maltreatment associated with adult personality disorders: findings from the collaborative longitudinal personality disorders study. *J. Pers. Disord.* 18 193–211. 10.1521/pedi.18.2.193.32777 15176757

[B8] Beebe B. Jaffe J. Markese S. Buck K. Chen H. Cohen P. (2010). The origins of 12-month attachment: a microanalysis of 4-month mother-infant interaction. *Attach. Hum. Dev.* 12 3–141. 10.1080/14616730903338985 20390524 PMC3763737

[B9] Beeney J. E. Wright A. C. Stepp S. D. Hallquist M. N. Lazarus S. A. Beeney J. S. (2016). Disorganized attachment and personality functioning in adults: a latent class analysis. *Pers. Disord.* 8 206–216. 10.1037/per0000184 26986959 PMC5026862

[B10] Bernard K. Dozier M. (2010). Examining infants’ cortisol responses to laboratory tasks among children varying in attachment disorganization: stress reactivity or return to baseline? *Dev. Psychol.* 46 1771–1778. 10.1037/a0020660 20873923 PMC3209263

[B11] Bernstein D. P. Fink L. (1998). *Childhood Trauma Questionnaire: A Retrospective Self-Report Manual.* Antonio, TX: The Psychological Corporation.

[B12] Bo S. Sharp C. Fonagy P. Kongerslev M. (2015). Hypermentalizing, attachment, and epistemic trust in adolescent BPD: clinical illustrations. *Pers. Disord.* 8 172–182. 10.1037/per0000161 26691672

[B13] Boldt L. J. Kochanska G. Yoon J. E. Nordling J. K. (2014). Children’s attachment to both parents from toddler age to middle childhood: links to adaptive and maladaptive outcomes. *Attach. Hum. Dev.* 16 211–229. 10.1080/14616734.2014.889181 24605850 PMC3997589

[B14] Bowlby J. (1980). *Attachment and Loss* 2nd Edn. New York, NY: Basic Books.

[B15] Bretherton I. (1993). “From dialogue to internal working models: The co-construction of self in relationships,” in *Memory and Affect in Development*, ed. Nelson C. A. (Hillsdale, NJ: Lawrence Erlbaum Associates, Inc.), 237–263.

[B16] Buchheim A. George C. (2011). “Attachment disorganization in borderline personality disorder and anxiety disorder,” in *Disorganized Attachment and Caregiving*, eds Solomon J. George C. (New York, NY: Guilford Press), 343–382.

[B17] Carlson E. A. Egeland B. Sroufe L. A. (2009). A prospective investigation of the development of borderline personality symptoms. *Dev. Psychopathol.* 21 1311–1334. 10.1017/S0954579409990174 19825270 PMC11034778

[B18] Chabrol H. Chouicha K. Montovany A. Callahan S. Duconge E. Sztulman H. (2002). Personality disorders in a nonclinical sample of adolescents. *L’Encéphale* 28 520–524. 12506264

[B19] Chanen A. M. Kaess M. (2012). Developmental pathways to borderline personality disorder. *Curr. Psychiatry Rep.* 14 45–53. 10.1007/s11920-011-0242-y 22009682

[B20] Chanen A. M. McCutcheon L. (2014). “Early intervention for borderline personality disorder,” in *Early Intervention in Psychiatry: EI of Nearly Everything for Better Mental Health*, eds Byrne P. Rosen A. Byrne P. Rosen A. (Wiley: Blackwell), 318–333.

[B21] Cicchetti D. Rogosch F. A. (2002). A developmental psychopathology perspective on adolescence. *J. Consult. Clin. Psychol.* 70 6–20. 10.1037/0022-006X.70.1.6 11860057

[B22] Dykas M. J. Woodhouse S. S. Ehrlich K. B. Cassidy J. (2012). Attachment-related differences in perceptions of an initial peer interaction emerge over time: evidence of reconstructive memory processes in adolescents. *Dev. Psychol.* 48 1381–1389. 10.1037/a0027462 22390661 PMC9364155

[B23] Finger B. Byun S. Melnick S. Lyons-Ruth K. (2015). Hostile-Helpless states of mind mediate relations between childhood abuse severity and personality disorder features. *Transl. Dev. Psychiatry* 3:28785 10.3402/tdp.v3.28785

[B24] Fonagy P. Target M. (1997). Attachment and reflective function: their role in self-organization. *Dev. Psychopathol.* 9 679–700. 10.1017/S0954579497001399 9449001

[B25] Fonagy P. Target M. Gergely G. Allen J. G. Bateman A. W. (2003). The developmental roots of borderline personality disorder in early attachment relationships: a theory and some evidence. *Psychoanal. Inq.* 23 412–459. 10.1080/07351692309349042

[B26] Fraley R. C. Roisman G. I. (2014). The adult attachment interview: psychometrics, stability and change from infancy, and developmental origins: III. Categories or dimensions? A taxometric analysis of the adult attachment interview. *Monogr. Soc. Res. Child Dev.* 79 36–50. 10.1111/mono.12112 25100088 PMC13222098

[B27] George C. Kaplan N. Main M. (1985). *Adult Attachment Interview.* Berkeley: University of California.

[B28] George C. Solomon J. (2008). “The caregiving system: A behavioral systems approach to parenting,” in *Handbook of Attachment: Theory, Research, and Clinical Applications*, 2nd Edn. eds Cassidy J. Shaver P. R. Cassidy J. Shaver P. R. (New York, NY: Guilford Press), 833–856.

[B29] Grienenberger J. F. Kelly K. Slade A. (2005). Maternal reflective functioning, mother-infant affective communication, and infant attachment: exploring the link between mental states and observed caregiving behavior in the intergenerational transmission of attachment. *Attach. Hum. Dev.* 7 299–311. 10.1080/14616730500245963 16210241

[B30] Gunderson J. G. (2007). Disturbed relationships as a phenotype for borderline personality disorder. *Am. J. Psychiatry* 164 1637–1640. 10.1176/appi.ajp.2007.07071125 17974925

[B31] Gunderson J. G. Lyons-Ruth K. (2008). BPD’s interpersonal hypersensitivity phenotype: a gene-environment-developmental model. *J. Pers. Disord.* 22 22–41. 10.1521/pedi.2008.22.1.22 18312121 PMC2596628

[B32] Ha C. Balderas J. C. Zanarini M. C. Oldham J. Sharp C. (2014). Psychiatric comorbidity in hospitalized adolescents with borderline personality disorder. *J. Clin. Psychiatry* 75 e457–e464. 10.4088/JCP.13m08696 24922498

[B33] Hesse E. Main M. (2006). Frightened, threatening, and dissociative parental behavior in low-risk samples: description, discussion, and interpretations. *Dev. Psychopathol.* 18 309–343. 10.1017/S0954579406060172 16600057

[B34] Holmes J. (2001). *The Search for the Secure Base: Attachment Theory and Psychotherapy.* New York, NY: Brunner-Routledge.

[B35] Holmes J. (2004). Disorganized attachment and borderline personality disorder: a clinical perspective. *Attach. Hum. Dev.* 6 181–190. 10.1080/14616730410001688202 15370510

[B36] Jacobsen T. Huss M. Fendrich M. Kruesi M. J. P. Ziegenhain U. (1997). Children’s ability to delay gratification: longitudinal relations to mother–child attachment. *J. Genet. Psychol.* 158 411–426. 10.1080/00221329709596679 9423273

[B37] Johnson D. M. Shea M. T. Yen S. Battle C. L. Zlotnick C. Sanislow C. A. (2003). Gender differences in borderline personality disorder: findings from the collaborative longitudinal personality disorders study. *Compr. Psychiatry* 44 284–292. 10.1016/S0010-440X(03)00090-7 12923706

[B38] Jones-Mason K. Allen I. E. Bush N. Hamilton S. (2016). Epigenetic marks as the link between environment and development: examination of the associations between attachment, socioeconomic status, and methylation of the SLC6A4 gene. *Brain Behav.* 6:e00480. 10.1002/brb3.480 27458544 PMC4951620

[B39] Kaess M. von Ceumern-Lindenstjerna I. Parzer P. Chanen A. Mundt C. Resch F. (2012). Axis I and II comorbidity and psychosocial functioning in female adolescents with borderline personality disorder. *Psychopathology* 46 55–62. 10.1159/000338715 22890504

[B40] Kaufman J. Birmaher B. Brent D. Rao U. (1997). Schedule for affective disorders and schizophrenia for School-Age Children-Present and Lifetime version (K-SADS-PL): initial reliability and validity data. *Journal of the American Academy of Child & Adolescent Psychiatry* 36 980–988. 10.1097/00004583-199707000-00021 9204677

[B41] Kaufman J. Birmaher B. Brent D. Rao U. Ryan N. (1996). *The Schedule for Affective Disorders and Schizophrenia for School-Age Children and Lifetime Version (Version 1.0).* Pittsburgh, PA: University of Pittsburgh School of Medicine.

[B42] Kernberg O. F. Yeomans F. E. Clarkin J. F. Levy K. N. (2008). Transference focused psychotherapy: overview and update. *Int. J. Psychoanal.* 89 601–620. 10.1111/j.1745-8315.2008.00046.x 18558958

[B43] Kobak R. Zajac K. Smith C. (2009). Adolescent attachment and trajectories of hostile-impulsive behavior: implications for the development of personality disorders. *Dev. Psychopathol.* 21 839–851. 10.1017/S0954579409000455 19583886 PMC4028819

[B44] Kochanska G. Kim S. (2013). Early attachment organization with both parents and future behavior problems: from infancy to middle childhood. *Child Dev.* 84 283–296. 10.1111/j.1467-8624.2012.01852.x 23005703 PMC3530645

[B45] Linehan M. M. (1987). Dialectical behavior therapy for borderline personality disorder: theory and method. *Bull. Menninger Clin.* 51 261–276. 3580661

[B46] Linehan M. M. (1993). *Cognitive-Behavioral Treatment of Borderline Personality Disorder.* New York, NY: Guilford Press.

[B47] Liotti G. (2004). Trauma, dissociation, and disorganized attachment: three strands of a single braid. *Psychotherapy* 41 472–486. 10.1037/0033-3204.41.4.472

[B48] Liotti G. (2014). “Disorganised attachment in the pathogenesis and the psychotherapy of borderline personality disorder,” in *Attachment Theory in Adult Mental Health: A Guide to Clinical Practice*, eds Danquah A. N. Berry K. Danquah A. N. Berry K. (New York, NY: Routledge/Taylor & Francis Group), 113–128.

[B49] Luijk M. M. Saridjan N. Tharner A. van IJzendoorn M. H. Bakermans-Kranenburg M. J. Jaddoe V. V. (2010). Attachment, depression, and cortisol: deviant patterns in insecure-resistant and disorganized infants. *Dev. Psychobiol.* 52 441–452. 10.1002/dev.20446 20583141

[B50] Lyons-Ruth K. (2008). Contributions of the mother-infant relationship to dissociative, borderline, and conduct symptoms in young adulthood. *Infant Ment. Health J.* 29 203–218. 10.1002/imhj.20173 19122769 PMC2613366

[B51] Lyons-Ruth K. Bronfman E. Parsons E. (1999). Maternal frightened, frightening, or atypical behavior and disorganized infant attachment patterns. *Monogr. Soc. Res. Child Dev.* 64 67–96. 10.1111/1540-5834.00034 10597543

[B52] Lyons-Ruth K. Bureau J. Holmes B. Easterbrooks A. Brooks N. H. (2013). Borderline symptoms and suicidality/self-injury in late adolescence: prospectively observed relationship correlates in infancy and childhood. *Psychiatry Res.* 206 273–281. 10.1016/j.psychres.2012.09.030 23123044 PMC3605274

[B53] Lyons-Ruth K. Holmes B. M. Henninghausen K. (2005). “Prospective longitudinal predictors of borderline and conduct symptoms in late adolescence: The early caregiving context,” in *Proceedings of the Borderline Psychopathology and Early Caregiving: Concurrent and Longitudinal Relations at the Biennial Meeting of the SOCIETY for Research in Child Development*, ed. Lyons-Ruth K. (Atlanta, GA).

[B54] Lyons-Ruth K. Melnick S. Patrick M. Hobson R. P. (2007). A controlled study of hostile-helpless states of mind among borderline and dysthymic women. *Attach. Hum. Dev.* 9 1–16. 10.1080/14616730601151417 17364479 PMC2585784

[B55] Madigan S. Moran G. Pederson D. R. (2006). Unresolved states of mind, disorganized attachment relationships, and disrupted interactions of adolescent mothers and their infants. *Dev. Psychol.* 42 293–304. 10.1037/0012-1649.42.2.293 16569168

[B56] Main M. (1995). “Recent studies in attachment: Overview, with selected implications for clinical work,” in *Attachment Theory: Social, Developmental, and Clinical Perspectives*, eds Goldberg S. Muir R. Kerr J. (Hillsdale, NJ: Analytic Press, Inc.), 407–474.

[B57] Main M. Goldwyn R. Hesse E. (2002). *Adult Attachment Scoring and Classification Systems. Version 7.1.* Berkeley: University of California at Berkeley.

[B58] Main M. Hesse E. (1990). “Parents’ unresolved traumatic experiences are related to infant disorganized attachment status: Is frightened and/or frightening parental behavior the linking mechanism,” in ed. Cummings E. M. (Chicago, IL: University of Chicago Press), 161–182.

[B59] Main M. Solomon J. (1986). “Discovery of an insecure-disorganized/disoriented attachment pattern,” in *Affective Development in Infancy*, eds Brazelton T. B. Yogman M. W. (Westport, CT: Ablex Publishing), 95–124.

[B60] Meins E. Fernyhough C. Wainwright R. Gupta M. D. Fradley E. Tuckey M. (2002). Maternal mind-mindedness and attachment security as predictors of theory of mind understanding. *Child Dev.* 73 1715–1726. 10.1111/1467-8624.00501 12487489

[B61] Miljkovitch R. (2009). *Les Fondations du Lien Amoureux.* Paris: Presses Universitaires de France.

[B62] Miljkovitch R. Moran G. Roy C. Jaunin L. Forcada-Guex M. Pierrehumbert B. (2013). Maternal interactive behaviour as a predictor of preschoolers’ attachment representations among full term and premature samples. *Early Hum. Dev.* 89 349–354. 10.1016/j.earlhumdev.2012.11.006 23265254

[B63] Miljkovitch R. Moss E. Bernier A. Pascuzzo K. Sander E. (2015). Refining the assessment of internal working models: the attachment multiple model interview. *Attach. Hum. Dev.* 17 492–521. 10.1080/14616734.2015.1075561 26325611

[B64] Normandin L. Ensink K. Kernberg O. F. (2015). Transference-focused psychotherapy for borderline adolescents: a neurobiologically informed psychodynamic psychotherapy. *J. Infant Child Adolesc. Psychother.* 14 98–110. 10.1080/15289168.2015.1006008

[B65] Ogawa J. R. Sroufe L. A. Weinfield N. S. Carlson E. A. Egeland B. (1997). Development and the fragmented self: longitudinal study of dissociative symptomatology in a nonclinical sample. *Dev. Psychopathol.* 9 855–879. 10.1017/S0954579497001478 9449009

[B66] Patrick M. Melnick S. Finger B. Hans S. Lyons-Ruth K. (2008). “Hostile-helpless states of mind in the AAI: A proposed additional AAI category with implications for identifying disorganized infant attachment in high-risk samples,” in *Clinical Applications of the Adult Attachment Interview*, eds Steele H. Steele M. (Guilford: Guilford Press), 399–423.

[B67] Peterson C. Slaughter V. (2003). Opening windows into the mind: mothers’ preferences for mental state explanations and children’s theory of mind. *Cogn. Dev.* 18 399–429. 10.1016/S0885-2014(03)00041-8

[B68] Pfohl B. Blum N. Zimmerman M. (1997). *Structured Interview for DSM-IV Personality: SIDP-IV.* Washington, DC: American Psychiatric Publishing.

[B69] Pilkonis P. A. Heape C. L. Ruddy J. Serrao P. (1991). Validity in the diagnosis of personality disorders: the use of the LEAD standard. *Psychol. Assess.* 3 46–54. 10.1037/1040-3590.3.1.46

[B70] Putnam K. M. Silk K. R. (2005). Emotion dysregulation and the development of borderline personality disorder. *Dev. Psychopathol.* 17 899–925. 10.1017/S0954579405050431 16613424

[B71] Rosenstein D. S. Horowitz H. A. (1996). Adolescent attachment and psychopathology. *J. Consult. Clin. Psychol.* 64 244–253. 10.1037/0022-006X.64.2.244 8871408

[B72] Schuppert H. M. Giesen-Bloo J. van Gemert T. G. Wiersema H. M. Minderaa R. B. Emmelkamp P. G. (2009). Effectiveness of an emotion regulation group training for adolescents—A randomized controlled pilot study. *Clin. Psychol. Psychother.* 16 467–478. 10.1002/cpp.637 19630069

[B73] Sharp C. Fonagy P. (2008). “Social cognition and attachment-related disorders,” in *Social Cognition and Developmental Psychopathology*, ed. Goodyer I. (New York, NY: Oxford University Press), 269–302.

[B74] Sharp C. Fonagy P. (2015). Practitioner review: borderline personality disorder in adolescence – recent conceptualization, intervention, and implications for clinical practice. *J. Child Psychol. Psychiatry* 56 1266–1288. 10.1111/jcpp.12449 26251037

[B75] Suess G. J. Grossmann K. E. Sroufe L. A. (1992). Effects of infant attachment to mother and father on quality of adaptation in preschool: from dyadic to individual organisation of self. *Int. J. Behav. Dev.* 15 43–65. 10.1177/016502549201500103

[B76] Trerotola M. Relli V. Simeone P. Alberti S. (2015). Epigenetic inhereritance and the missing heritability. *Hum. Genom.* 28:17 10.1186/s40346-015-0041-3 PMC4517414 26216216

[B77] Trull T. J. (2015). “Borderline personality disorder: Contemporary approaches to conceptualization and etiology,” in *Oxford textbook of psychopathology*, 3rd Edn. eds Blaney P. H. Krueger R. F. Millon T. Blaney P. H. Krueger R. F. Millon T. (New York, NY: Oxford University Press),768–790.

[B78] Tyrer P. (2014). “The likely classification of borderline personality disorder in adolescents in ICD-11,” in *Handbook of Borderline Personality Disorder in Children and Adolescents*, eds Sharp C. Tackett J. L. Sharp C. Tackett J. L. (New York, NY: Springer Science + Business Media),451–457.

[B79] Verschueren K. Marcoen A. (1999). Representation of self and socioemotional competence in kindergartners: differential and combined effects of attachment to mother and father. *Child Dev.* 70 183–201. 10.1111/1467-8624.00014 10191522

[B80] Zanarini M. C. Frankenburg F. R. Reich D. B. Marino M. F. Lewis R. E. Williams A. A. (2000). Biparental failure in the childhood experiences of borderline patients. *J. Pers. Disord.* 14 264–273. 10.1521/pedi.2000.14.3.264 11019749

[B81] Zanarini M. C. Williams A. A. Lewis R. E. Reich R. B. Vera S. C. Marino M. F. (1997). Reported pathological childhood experiences associated with the development of borderline personality disorder. *Am. J. Psychiatry* 154 1101–1106. 10.1176/ajp.154.8.1101 9247396

